# Implementation of a nursing assessment framework in the postanesthesia care unit: a pre-post implementation design

**DOI:** 10.3389/fmed.2026.1849111

**Published:** 2026-07-01

**Authors:** Ping Bai, Guozheng Chen, Xianxian Zang, Caiyun Li, Ziyi Zhang, Lu Wang, Guoyong Yang

**Affiliations:** 1 Postanesthesia Care Unit, Department of Oral and Maxillofacial Surgery, Peking University School and Hospital of Stomatology & National Center of Stomatology & National Clinical Research Center for Oral Diseases & National Engineering Research Center of Oral Biomaterials and Digital Medical Devices, Beijing, China; 2 School of Nursing, Peking University, Beijing, China; 3 Operating Room, Department of Oral and Maxillofacial Surgery, Peking University School and Hospital of Stomatology & National Center of Stomatology & National Clinical Research Center for Oral Diseases & National Engineering Research Center of Oral Biomaterials and Digital Medical Devices, Beijing, China

**Keywords:** Anesthesia Recovery Period, nursing assessment, patient safety, postanesthesia nursing, quality improvement

## Abstract

**Introduction:**

Structured nursing assessment during the postanesthesia period is critical for patient safety, yet assessment practices in the postanesthesia care unit (PACU) often remain inconsistent. The Nursing Assessment Framework for Patients in the Anesthesia Recovery Period (NAF-PARP) was previously developed, but its implementation and impact have not been evaluated. This study aimed to implement NAF-PARP in a PACU and assess its effects on nursing assessment quality and the incidence of nursing defects.

**Methods:**

This study used a pre- and post-intervention quality improvement design. A total of 90 patients admitted between September and December 2024 were assessed using the existing model (control group). After a 2-month training program for nurses (January–February 2025), a second group of 90 patients admitted between March and June 2025 was assessed using NAF-PARP (intervention group). Outcome measures included scores for different assessment domains and the incidence of nursing defects.

**Results:**

Improvements were observed in physical assessment, clinical judgment, and organizational efficiency in the intervention group. However, there was no significant difference in the incidence of nursing defects between the two groups.

**Conclusion:**

Implementation of NAF-PARP was associated with improvements in key domains of nursing assessment quality but did not reduce nursing defects. These findings support the value of structured assessment frameworks in postanesthesia nursing while highlighting the complexity of translating process improvements into patient safety outcomes. Further research is warranted to explore longer-term effects and system-level strategies.

## 1 Introduction

The postanesthesia period associated with general anesthesia represents a critical juncture in the surgical journey. It begins with the cessation of anesthetic administration and extends until patients regain full consciousness and physiological stability, at which point they are safely discharged from the postanesthesia care unit (PACU) (1). During this vulnerable phase, patients face heightened risks of severe complications due to factors such as compromised physiological reserves, imbalanced homeostasis, surgical stress responses, uncontrolled bleeding, and residual anesthetic effects (2). Consequently, comprehensive assessment and timely intervention are essential to anticipate and mitigate postoperative complications during this critical period.

Nursing assessment is a systematic process that involves identifying patients' physical and psychosocial needs as well as current or potential health problems, and determining how those needs can be addressed through systematic information collection and analysis (3). Comprehensive postanesthesia assessment should encompass multiple domains, such as respiratory and cardiovascular function, neuromuscular status, mental status, temperature, pain, nausea and vomiting, fluid balance, urine output, voiding, drainage, and bleeding (4).

Despite the established importance of these assessment domains, their actual execution in clinical practice remains inconsistent. Research indicates that assessments of newly admitted patients—as well as ongoing assessments throughout the PACU stay—are frequently omitted, potentially leading to delayed recognition of clinical deterioration and adverse patient outcomes (5). Such omissions contribute to nursing defects and may adversely affect patient recovery (6). This inconsistency underscores the need for standardized approaches to strengthen assessment practices among PACU nurses.

Existing instruments for PACU assessment have primarily focused on discharge readiness. Scoring systems such as the Aldrete Score, Modified Aldrete Score, and White Scoring System provide structured criteria for determining when patients meet safety thresholds for PACU discharge (7). While these tools have contributed to standardization of discharge decisions, they are limited in scope—designed specifically for discharge readiness rather than comprehensive, ongoing assessment throughout the patient's PACU stay. Moreover, these existing systems do not address the broader cognitive, procedural, and humanistic dimensions of nursing assessment that are essential for patient care during the postanesthesia period. Cenacchi et al. and Giusti et al. highlighted that organizational reforms in staffing and resource integration are essential for improving clinical outcomes (8, 9). Informed by this, we implemented a framework as part of a broader organizational change including training, leadership reinforcement, and peer support.

To address this gap, Peng et al. (10) developed a novel nursing assessment framework specifically designed for patients during the postanesthesia period. They constructed the Nursing Assessment Framework for Patients in the Anesthesia Recovery Period (NAF-PARP), which provides a cognitive workflow structure to guide nurses' assessment practice. The framework is grounded in the mini-clinical evaluation exercise (mini-CEX). NAF-PARP was designed to focus on the comprehensive, ongoing assessment process throughout the patient's PACU stay. Given the fragmented nature of PACU assessments due to time pressure and competing priorities, structured frameworks that help organize and prioritize assessment information may be particularly valuable. Similar frameworks and assessment tools have indicated positive effects on nurses' clinical decision-making abilities and clinical competence. Hvidberg et al. (11) highlighted the importance of structured reasoning in PACU discharge decision-making. These findings suggest the potential value of such structured approaches in nursing practice (11).

A pre-implementation contextual assessment was conducted to identify local barriers and facilitators (see Methods Section).

The present quality improvement (QI) initiative aims to integrate the NAF-PARP in a clinical PACU setting and evaluate its effects on nursing assessment quality and the incidence of nursing defects. Guided by Rogers' Diffusion of Innovations Theory, this study also examines the adoption process of the framework among PACU nurses, providing insights into how structured assessment tools can be effectively integrated into routine clinical practice.

## 2 Methods

### 2.1 Design

This QI study employed a pre- and post-implementation design.

### 2.2 Study setting and sampling

This study was conducted in the PACU of a university-affiliated tertiary stomatological hospital in China. Patients admitted to the PACU primarily underwent oral and maxillofacial surgeries. At the time of the study, the eight-bed PACU was staffed by 14 full-time nurses who provided care for approximately 7,000 patients annually. In addition to postanesthesia nursing, the PACU also provided critical care services.

This study focused on the nursing assessment process performed by PACU nurses. To avoid bias related to knowledge and clinical experience, 12 nurses with 3 or more years of experience in the PACU were included. Throughout the study period, the nursing staff remained stable, with no personnel changes between the pre- and post-implementation phases.

Sample size was estimated using G^*^Power software. The primary outcome measure was nursing assessment scores, which were compared before and after the intervention. An independent samples *t*-test was used, with a medium effect size (Cohen's *d* = 0.5), a significance level of α = 0.05, and a power of 1–β = 0.90. A 1:1 allocation ratio yielded a minimum of 86 patients per group. A total of 180 observations were conducted, with each of the 12 nurses observed seven to eight times before and after the intervention (90 patients per period). For confidentiality purposes and because this study was not designed to evaluate individual nurse performance, nurses' names were not recorded, and their scores were not linked to individual identifiers.

### 2.3 Contextual assessment

Prior to this initiative, a review of incident reports and nursing documentation revealed inconsistent assessment practices. During the 6-month period from January to June 2024, an average of 3.2 required assessment items per patient were documented as incomplete or omitted, and two near-miss events related to delayed recognition of postoperative oxygen desaturation were attributed to incomplete assessments. To systematically identify barriers and facilitators, we conducted a pre-implementation contextual assessment using three data sources: (a) retrospective review of incident reports (*n* = 6 months); (b) two informal staff meetings with content documented by the head nurse; and (c) a brief written survey of all 12 nurses' perceptions (five-item questionnaire covering time pressure, cognitive guidance, handover variability, leadership support, and willingness to change). Quantitative survey data were summarized using frequencies, while qualitative comments from meetings and open-ended survey responses were analyzed using thematic synthesis. The main barriers identified were: lack of a cognitive guide (reported by 10/12 nurses), time pressure during high-volume admissions (11/12), and variability in handover content (9/12). Facilitators included strong head nurse leadership (12/12) and staff willingness to improve (10/12). These local data underscored the need for a standardized approach.

### 2.4 Study processes and patients

A 4-month period (September to December 2024) was used to observe and evaluate the quality of nursing assessments using the existing approach. Subsequently, a 2-month period (January and February 2025) was dedicated to applying the QI process, during which the new nursing assessment framework was introduced. The subsequent 4 months (March to June 2025) were then used to evaluate the impact of this new framework.

A convenience sampling method was used to select patients for observation in the postanesthesia period during two designated time periods. The primary unit of analysis was each patient assessment event. Inclusion criteria were: (a) aged 18 years or older; (b) American Society of Anesthesiologists (ASA) physical status classification I–III; (c) admitted to the PACU with an endotracheal tube in place and expected to be extubated and regain consciousness within 2 h; and (d) provision of informed consent. Patients were excluded if they experienced delayed awakening or developed severe complications requiring rescue interventions that exceeded the routine nursing intensity of the PACU. ASA IV-V patients were excluded because their unpredictable, prolonged stays and frequent rescue interventions could confound routine PACU assessment measures.

### 2.5 The QI team

The QI team was explicitly constituted to ensure clear roles and responsibilities throughout the implementation process. The team was led by the PACU nurse manager (head nurse) and two charge nurses, who coordinated the overall process, gathered feedback, and facilitated communication between team members. All 12 PACU nurses were involved as end-users of the framework. Two co-first authors of this study (Bai and Chen) served as independent observers and evaluators, without participating in direct patient care. Three early adopters, selected based on their clinical expertise and active engagement during training, acted as peer role models during the implementation phase. Anesthesiologists and surgeons were informed about the initiative but were not directly involved, as the intervention focused on nursing assessment processes. Patients were not part of the design team due to postoperative communication difficulties.

### 2.6 The QI initiative

The successful implementation of a new assessment framework depends not only on its clinical value but also on nurses' acceptance and adoption of it. Therefore, a theoretical approach is needed to guide the implementation process. The design and implementation of this QI initiative were informed by Rogers' Diffusion of Innovations Theory. This theory posits that the adoption of an innovation is influenced by its perceived attributes: relative advantage, compatibility, complexity, trialability, and observability (12, 13). This theory provides a well-established framework for understanding how innovations are introduced, adopted, and sustained within organizations. In this study, the authors applied this framework to examine the adoption of NAF-PARP among PACU nurses. Specifically, the study sought to: (1) evaluate the framework's relative advantage compared to traditional assessment practices; (2) assess its compatibility with existing clinical workflows; and (3) explore its initial adoption and diffusion within the nursing team. Based on the theory's emphasis on the role of early adopters, the intervention strategies were designed to enhance the framework's perceived advantages and ease of use, thereby facilitating engagement among frontline nurses.

According to Rogers' Theory, adoption among individuals consists of five stages: knowledge, persuasion, decision, implementation, and confirmation. These phases served to guide the design of implementation activities, including educational strategies, peer support, pilot testing, feedback mechanisms, and reinforcement strategies.

#### 2.6.1 Knowledge

The knowledge stage is when individuals first learn about an innovation and how to use it (14). To help nurses adopt NAF-PARP, all PACU nurses attended a 1-h session comprising a 30-min slide presentation and a 30-min simulation. Sessions were offered within the first 2 weeks of the study period, allowing nurses to choose a time that fit their shifts. Training emphasized the validity and ease of use of NAF-PARP. During simulations, nurses practiced with standardized patient scenarios, completed the structured template, and received immediate feedback. This addressed concerns about the time needed to complete the assessment. To reinforce daily use, a printed NAF-PARP template was placed at each bedside.

#### 2.6.2 Persuasion

In the persuasion stage, individuals develop a more positive attitude toward the innovation, and favorable views spread among potential adopters. Influencers include peers, senior nurses, and opinion leaders such as head nurses (15). Their role is crucial during early adoption (16). Through social influence and role modeling, external persuasion becomes internalized. Individual reflection on the innovation's fit and usefulness also plays a key role (17). Persuasion thus involves both social and cognitive processes.

In this study, the PACU head nurse, as QI team leader, encouraged adoption. During daily informal interactions, she inquired about initial experiences, addressed concerns, and gave positive feedback. A pilot was first conducted with charge nurses, after which early adopters were selected to promote acceptance. These early adopters acted as visible role models. In daily practice, they verbalized their thought processes while using NAF-PARP, making the framework observable. During breaks, they shared how the framework helped prioritize assessments and reduce omissions. This peer-driven approach leveraged social influence. A 1-week “trial period” allowed nurses to try the framework without formal evaluation, so they could assess its compatibility with their workflow.

#### 2.6.3 Decision

After persuasion, individuals decide whether to adopt or reject the innovation. They often seek additional information to reduce uncertainty and validate their attitudes (18). Reinforcing perceived benefits and compatibility is key. To support this, the head nurse regularly emphasized correct use and precautions of NAF-PARP during weekly post-shift seminars. She also presented pilot data showing improved assessment completeness among early adopters. Each seminar ended with a Q&A session, where nurses raised concerns (e.g., handling complex patients or time constraints) and received immediate clarification. A one-page FAQ sheet was created and shared with all nurses.

#### 2.6.4 Implementation

During this stage, nurses used NAF-PARP as a cognitive framework throughout patient assessment. To ensure safe adoption, nurses were paired by seniority: junior nurses worked alongside senior nurses, who served as “go-to” resources. Junior nurses were encouraged to consult their senior partners when uncertain. Weekly reflective seminars promoted peer learning. Nurses presented real clinical cases, discussed successes and difficulties, and collaboratively exchanged solutions. A “challenge log” on a whiteboard allowed anonymous reporting of obstacles, which were addressed in seminars or via follow-up. A quick-reference pocket card summarizing NAF-PARP was given to each nurse.

#### 2.6.5 Confirmation

In the final stage, individuals seek reinforcement for their adoption decision and recognize the innovation's value. Peer and opinion leader support is crucial to validate decisions and sustain long-term adoption. We used multiple reinforcement strategies. First, positive pilot results were shared in weekly seminars. A poster comparing pre- and post-implementation assessment quality indicators was displayed in the break room. Nurses with exemplary use were informally acknowledged by the head nurse during shift huddles. Second, clinical educators and early adopters continued to model NAF-PARP daily and offered informal encouragement. Four months after implementation, a follow-up survey showed that 92.9% of nurses remained satisfied; these findings were shared as evidence of continued positive reception. These strategies helped nurses internalize NAF-PARP, enabling more comprehensive patient assessment and critical self-reflection.

These five stages, along with their corresponding core activities and key outputs, are summarized in Table 1.

**Table 1:** Five-stage implementation of NAF-PARP: core activities and key outputs.

Phase	Core activities	Key outputs
**Knowledge**	• 1-h training • Scenario simulation • Bedside template	• Framework awareness • Basic skills • Time concerns solved
**Persuasion**	• Leader support • Peer modeling • 1-week trial	• Positive attitudes • Peer influence • Personal fit checked
**Decision**	• Weekly data seminars • Q&A sessions • FAQ handout	• Less uncertainty • Advantages seen • Adoption decided
**Implementation**	• Senior–junior pairing • Weekly case reviews • Pocket card	• Framework used daily • Challenges tackled • Peer network formed
**Confirmation**	• Results poster • Peer recognition • Ongoing modeling	• Value accepted • Framework internalized • Reflection improved

### 2.7 Measurement and quality control

#### 2.7.1 Quality of nursing assessment

Two co-first authors of this study served as observers and evaluators throughout the QI period. Both were present simultaneously at each observation session to assess nurses' performance using the NAF-PARP framework. The observers did not participate in patient care or the assessment process, serving solely as nonparticipant observers to minimize interference with clinical practice. Throughout the study period, all nurses were aware of the observers' presence during the assessment process.

While NAF-PARP was designed to guide assessment practice, its itemized structure also enabled systematic evaluation of assessment quality (10). The framework is based on the mini-clinical evaluation exercise (mini-CEX) format and consists of five domains encompassing a total of 24 items (19). The history-taking domain includes five items: patient identification, surgical condition, anesthesia status, intraoperative nursing care, and drugs/blood products. The physical assessment domain includes five items: surgical wounds, specialty assessment, lines/drains, skin condition, and positioning. The clinical judgment domain includes nine items: consciousness, respiration, circulation, temperature, airway, drug effects, pain, muscle status, and nausea/vomiting. The organizational efficiency domain includes two items: timely response and priority handling. The humanistic concern domain includes three items: privacy protection, comfort, and injury prevention. Consistent with the mini-CEX scoring scheme, each item is rated on a 10-point scale (0–10), with higher scores indicating better performance in the corresponding aspect of nursing assessment. The total possible score for the NAF-PARP is 240. Domain scores are calculated by summing the scores of all items within that domain, and the overall scale score is the sum of the five domain scores (10). This framework served as both the cognitive guide for nurses' assessment practice and the evaluation tool for measuring assessment quality in this study.

For each observed case, both observers independently completed the NAF-PARP scoring form based on their assessment of the nurse's performance throughout the entire patient assessment process. Each observation session lasted approximately 30–60 min, covering the complete assessment process from patient admission through ongoing monitoring. Item scores were recorded for each of the 24 items, and domain scores and total scores were subsequently computed by summation. The final score was calculated as the average of the two observers' ratings.

Prior to formal data collection, a pilot test was conducted to establish inter-rater reliability. Ten patient assessment cases were independently observed and rated by both observers. Prior to the pilot test, the two observers discussed the scoring criteria to calibrate their understanding. During the pilot test, each observer completed the scoring forms independently without discussion. After completing all ten observations, the two observers met again to review their ratings and discuss any discrepancies, further refining their alignment. The inter-rater reliability coefficient (Pearson's correlation) was 0.86 (*P* < 0.01), indicating a high level of consistency between the two observers' ratings. This acceptable level of agreement supported the reliability of subsequent data collection.

To minimize potential bias, observers did not have access to nurses' prior performance scores and refrained from discussing their ratings with each other during individual observation sessions. In addition, to reduce the potential bias that may arise from a single nurse being observed for multiple patients, only one nurse-patient pair was observed per day. All observation records were anonymized, with nurses identified only by unique study codes, and no individual performance data were shared with unit managers or incorporated into staff evaluations.

#### 2.7.2 Incidence of nursing defects

The incidence of nursing defects was assessed through nursing records and the hospital's adverse event reporting system. Nursing defects were defined as deviations from standard care and were further classified into five categories: (1) assessment errors (e.g., inaccurate vital sign measurement and inappropriate timing of assessment); (2) documentation omissions or inaccuracies (e.g., missing records, discrepancies with actual conditions); (3) patient safety risk events (e.g., delayed recognition of clinical deterioration); (4) nursing-related adverse events that led or could have led to patient harm (e.g., medication errors and unplanned tube removal); and (5) other deviations not covered by the above.

For both 4-month periods, two authors independently reviewed all PACU patient nursing records and retrieved all related adverse event reports. All patients admitted during these periods were included—those in the preimplementation period assessed with the traditional approach, and those in the post-implementation period assessed with NAF-PARP.

To ensure consistent classification, a structured data collection form was developed specifically for this study. The two reviewers were blinded to the study period during data extraction and classification. Discrepancies were resolved through discussion until consensus was reached. Cross-verification between nursing records and adverse event reports was performed to ensure completeness, with duplicate events counted only once. The defect rate for each period was calculated as the number of confirmed defect cases divided by the total number of patients admitted, expressed as a percentage. No nurse identifiers were recorded.

#### 2.7.3 Contextual and other factors

The contextual factors that may have influenced the adoption and effectiveness of NAF-PARP were informally monitored through weekly seminar discussions and the challenge log. Factors such as staffing levels, patient acuity, and unit workload were noted but not systematically measured.

Due to the relatively short observation period (4 months per phase) and the stable context of the unit, temporal trends were not formally analyzed.

Throughout the whole process of this QI Period, any unintended consequences, including patient harm or other unanticipated outcomes, were documented in detail. These events were then investigated to explore potential solutions.

### 2.8 Statistical analysis

Statistical analyses were performed using SPSS version 26.0 (IBM Corp., Armonk, NY, USA). Descriptive statistics were used to summarize patient demographic and surgical characteristics, nursing assessment scores, and the incidence of nursing defects. Continuous variables were presented as means with standard deviations (SD) or medians with interquartile ranges (IQR), as appropriate. Categorical variables were expressed as frequencies and percentages.

For inferential analysis, independent samples *t*-tests were employed to compare continuous variables between the pre- and post-implementation groups, including nursing assessment domain scores. Pearson's chi-square tests were used to examine differences in categorical variables, such as patient characteristics and the incidence of nursing defects. All statistical tests were two-tailed. Given the exploratory nature of this QI initiative and the absence of nurse identifiers, the *t*-tests were used for descriptive comparison. The primary evidence for improvement is based on the magnitude of the effect sizes (Cohen's *d*) and the consistent pattern of changes across domains, rather than on *p*-values alone.

### 2.9 Ethical considerations

This project was reviewed by the Institutional Review Board of Peking University School and Hospital of Stomatology and deemed exempt from formal ethics approval, as it constituted a quality improvement initiative. The project was approved by the head of the clinical unit in which it was conducted. All participating patients were informed about the study prior to surgery, and their written informed consent was obtained before inclusion. Verbal consent was obtained from all participating nurses. To protect privacy, all written observation records were anonymized and did not contain any personal identifiers for either patients or nurses. The study was conducted in accordance with the 1964 Helsinki declaration and its later amendments.

This manuscript is reported in accordance with the Standards for Quality Improvement Reporting Excellence (SQUIRE 2.0) guidelines (20).

## 3 Results

### 3.1 General information of patients

A total of 180 patients were observed across the two study periods, with no cases excluded due to any reason. Patient age ranged from 18 to 81 years, with a mean of 45.36 years. Females slightly outnumbered males. Patients with an American Society of Anesthesiologists (ASA) physical status classification of I accounted for 65.6% of the sample. Detailed characteristics are presented in Table 2.

**Table 2:** Demographic and clinical characteristics of patients.

&Variable	Control group *&n* = &90		Intervention group *&n* = &90		Chi^2^/*t*	*P*
Age (mean, SD)	46.27	14.50	44.44	14.91	0.831^a^	0.407
Gender (*n*, %)					0.561	0.454
Male	38	42.2%	43	47.8%		
Female	52	57.8%	47	52.2%		
ASA classification (*n*, %)					1.034	0.596
I	62	68.9%	56	62.2%		
II	21	23.3%	24	26.7%		
III	7	7.8%	10	11.1%		

^a^t value.ASA, American Society of Anesthesiologists; SD, Standard deviation.

### 3.2 Quality of nursing assessment scores

Prior to introduction of the framework, the mean total nursing assessment score was 147.01, which increased to 183.26 post-implementation (*P* < 0.001). Furthermore, compared with preimplementation results, the improvements observed in the physical assessment, clinical judgement, and organizational efficiency domains were also statistically significant (*P* < 0.001). Detailed results are shown in Table 3.

**Table 3:** Nursing assessment scores pre- and post-implementation.

Domain	Number of items	Pre		Post		Mean difference (post–pre)	*t*	95% CI	Cohen's *d*	*P*
		Mean	SD	Mean	SD			
History taking	5	37.82	4.01	38.76	3.42	0.94	1.78	−0.10–1.98	0.27	0.076
Physical assessment	5	27.54	4.45	40.27	3.08	12.73	23.52	11.66–13.80	2.49	< 0.001
Clinical judgement	9	50.62	9.58	69.54	7.78	18.92	15.34	16.48–21.36	2.29	< 0.001
Organizational efficiency	2	10.41	3.32	13.45	2.74	3.04	7.06	2.19–3.89	0.99	< 0.001
Humanistic concern	3	20.62	3.70	21.24	3.07	0.62	1.29	−0.34 to 1.58	0.19	0.199
Total scores	24	147.01	13.08	183.26	10.43	36.25	21.67	33.03–39.47	2.30	< 0.001

SD, Standard deviation.

### 3.3 Incidence of nursing defect

During the two study periods, a total of 2,609 and 2,647 patients were admitted to the PACU before and after implementation, respectively. A total of 50 nursing defects were identified, with 22 occurring in the preimplementation period and 28 in the post-implementation period. A comparison of the incidence rates is presented in Table 4.

**Table 4:** Incidence of nursing defects before and after implementation.

Variables	Pre (*n* = 2,609)	Post (*n* = 2,647)	Chi^2^	*P*
Nursing defects, *n* (%)	22 (0.84)	28 (1.06)	0.64	0.423

### 3.4 Implementation process summary

The implementation of NAF-PARP followed Rogers' five stages between January and June 2025. All 12 PACU nurses completed the 1-h training. After a 1-week trial period, the framework was fully adopted in daily practice. A total of 180 patient assessments were observed (90 preimplementation, 90 post-implementation), with each nurse observed seven to eight times per phase. No protocol deviations or adverse events related to the implementation were recorded.

### 3.5 Contextual observations and unintended consequences

Throughout the whole period, informal monitoring through weekly seminar discussions and the challenge log revealed no significant fluctuations in contextual factors such as staffing levels, patient acuity, or unit workload that would have influenced the adoption of NAF-PARP. The unit environment remained stable throughout both study phases.

Weekly seminar discussions suggested that assessment performance strengthened gradually over the implementation period, with later observations showing greater consistency among nurses. This qualitative observation aligns with the improvements in assessment scores reported in Section 3.2.

No unintended consequences—including patient harm or other unanticipated outcomes—were documented during the QI process. The challenge log and weekly seminars did not reveal any unanticipated problems associated with the framework.

## 4 Discussion

This QI initiative highlighted that implementation of the NAF-PARP was associated with improvements in nursing assessment performance, particularly in the domains of physical assessment, clinical judgement, and organizational efficiency. However, no considerable changes were observed in health history taking or humanistic concern, and the incidence of nursing defects did not change significantly following implementation. These findings suggest that while structured cognitive frameworks can effectively enhance certain dimensions of nursing assessment, their impact on patient safety outcomes may be more complex and warrant further investigation.

### 4.1 Mechanisms underlying the improvement in nursing assessment scores

The observed improvements in assessment performance can be attributed to three interrelated mechanisms: enhanced understanding of assessment priorities, improved application of assessment principles into clinical practice, and increased consistency in assessment practices across nurses.

#### 4.1.1 Understanding

Structured training using NAF-PARP advanced nurses' assessment performance, consistent with evidence that competency-based interventions enhance clinical knowledge and practice behaviors (21). However, these improvements extend beyond individual skill acquisition to encompass cognitive and organizational mechanisms. Framework-based training helped nurses bring their tacit clinical knowledge to the surface and shape it into explicit, shared cognitive schemas (22). This collective externalization promoted shared understanding of what constitutes comprehensive PACU assessment. Thus, NAF-PARP functioned not merely as a knowledge delivery tool, but as a cognitive scaffold enabling nurses to articulate and internalize assessment knowledge collectively (23).

This enhanced understanding was most evident in physical examination performance. In PACU settings, assessment fragmentation often stems from contextual constraints—time pressure, high task density, environmental noise—rather than deficiencies in clinical knowledge (6). NAF-PARP training enabled nurses to reorganize dispersed examination cues into explicit, sequential assessment schemas. Consequently, strengthened physical examination scores reflect not only increased examination frequency, but deeper understanding of assessment priorities and clinical relevance during recovery (24). From Rogers' diffusion theory perspective, such cognitive structuring reduces perceived complexity and enhances trialability of the assessment process (25).

#### 4.1.2 Application

Enhanced understanding alone does not automatically translate into reinforced practice unless operationalized into concrete assessment behaviors (26). In PACU environments, nurses must rapidly transform cognitive awareness into timely decisions and coordinated actions (11). Observed improvements suggest that NAF-PARP enabled nurses to bridge the gap between knowing what to assess and effectively enacting assessments within clinical workflows.

This application process was most evident in enhanced clinical judgement. Reinforced clinical judgement reflects nurses' growing capacity to translate assessment priorities into enacted decisions guided by an explicit framework, reducing reliance on individual preference or situational improvisation (27). Consequently, deviations in judgement processes across nurses were attenuated, and assessment omissions due to discretionary decision-making were reduced (28). Thus, improved clinical judgement represents a critical mechanism through which individualized understanding was translated into standardized, reproducible assessment behaviors.

#### 4.1.3 Consistency

Building on enhanced understanding and application, strengthened consistency emerged in assessment practices. Consistency refers to alignment of assessment priorities, decision pathways, and response patterns across nurses and clinical situations—particularly critical in PACU settings where patient conditions progress rapidly and care continuity depends on coordinated team performance (29). Observed improvements suggest NAF-PARP reduced unwarranted variation and promoted predictable, reproducible assessment practices.

Improved organizational efficiency represents a concrete manifestation of enhanced consistency. Higher scores indicate nurses were increasingly aligned in responding to patient requirements (10). Through NAF-PARP training, organizational expectations were clarified and shared, enabling timely recognition of patient needs across the nursing team (30). Enhanced organizational efficiency thus reflects both improved efficiency and a shared, priority-driven approach to patient assessment.

Notably, improvements aligned closely with NAF-PARP training objectives—promoting timely, priority-oriented assessment. The fact that improvements were most evident in targeted domains (prioritized decision-making, timely responses) suggests observed consistency reflects deliberate translation of training intent into practice outcomes, reinforcing the validity of the consistency improvements identified (31).

### 4.2 Incidence of nursing defects

For all the gains in assessment quality, nursing defect rates held steady. Several factors may account for this apparent disconnect.

One reason lies in the nature of nursing defects themselves: they are low-frequency events shaped by far more than assessment practices alone (32). Improvements in assessment quality are best understood as proximal outcomes, while measurable reductions in defects represent distal safety outcomes (33). Enhanced assessment processes are necessary for patient safety, but they cannot single-handedly deliver immediate reductions in adverse events (34). True safety gains typically require a constellation of reinforcing strategies—adequate staffing, strong communication, engaged leadership, and a robust safety culture (35).

Timing also matters. Behavioral changes take time to embed, and heightened awareness can paradoxically inflate defect reports before reducing them. Training interventions require sustained reinforcement to become fully integrated into routine practice (26). Moreover, sharper situational awareness and more rigorous assessment may initially surface more potential defects, masking any true reduction in incident rates during early implementation. Short observation windows can easily underestimate the downstream impact of process-oriented interventions (36). Consequently, the absence of a significant reduction in nursing defect rates in this study was not entirely unexpected given the relatively short follow-up period.

Then there is the measurement challenge. With low baseline rates and reliance on incident reports, subtle improvements are easily missed. Defect rates in PACU settings are typically low to begin with, making statistical detection of change difficult within limited samples and timeframes. And incident reporting systems are prone to underreporting and bias, further dulling their sensitivity to change (37). These measurement characteristics constrain the ability to capture subtle or incremental safety improvements attributable to assessment-focused interventions.

Collectively, these findings suggest that while NAF-PARP meaningfully improved assessment practice, reducing defect rates will likely demand a broader, system-level effort. Future initiatives should pair assessment optimization with complementary strategies—longitudinal reinforcement, interdisciplinary coordination, and organizational safety culture change. Only such a multifaceted, sustained approach may ultimately translate process improvements into measurable gains in patient safety.

### 4.3 Strengths and limitations

This study offers several distinctive strengths. Rather than adding another checklist to PACU care, it focused on reshaping how nurses think through patient assessment. This emphasis on cognitive structuring represents a novel approach to improving nursing assessment quality in the PACU setting. The study also turned its lens on nurses themselves, examining how assessment priorities are internalized and enacted in real time. Structured training and sustained reinforcement allowed the framework to take root in daily workflows, with clear gains in assessment performance. Complementing this focus on nurses was the training approach itself. Its blend of didactic instruction, hands-on simulation, and iterative feedback proved its value across domains. The intervention required dedicated time for training (1 h per nurse), weekly seminars (30 min per week), and ongoing reinforcement by the head nurse and early adopters. These investments were accommodated within existing shift schedules without additional staffing costs, suggesting acceptable opportunity costs for the observed improvements.

Several limitations warrant consideration. As a single-center study restricted to ASA I–III patients, our findings may not be generalizable to other PACU settings or more medically complex populations. The pre-post design without a concurrent control group limits causal inference; observed changes could reflect secular trends, Hawthorne effects, or unmeasured confounders. Methodologically, treating repeated observations from the same nurses as independent may have introduced clustering bias, and using NAF-PARP as both the intervention and outcome tool could have caused measurement bias. Future studies should employ multilevel modeling and separate instruments for intervention and outcome assessment. Finally, this study tested implementation and impact, not validation of the NAF-PARP itself.

## 5 Conclusion

This QI initiative showed that implementing NAF-PARP in the PACU was associated with improvements in physical assessment, clinical judgement, and organizational efficiency. These gains suggest that structured frameworks may strengthen assessment quality during recovery. However, nursing defect rates did not change, indicating that process improvements alone may not quickly translate into safety gains. The framework was integrated into daily practice, and the observed improvements support its potential role in standardizing postanesthesia care. Future work should include larger, multicenter samples and longer follow-up to assess whether such frameworks ultimately improve patient outcomes across varied settings.

## Data Availability

The raw data supporting the conclusions of this article will be made available by the authors, without undue reservation.
